# Oxidative stress mediates end-organ damage in a novel model of acetaminophen-toxicity in *Drosophila*

**DOI:** 10.1038/s41598-022-21156-w

**Published:** 2022-11-11

**Authors:** Bejan J. Saeedi, Sarah Hunter-Chang, Liping Luo, Kaiyan Li, Ken H. Liu, Brian S. Robinson

**Affiliations:** 1 Department of Pathology, Emory University School of Medicine, 615 Michael Street, Atlanta, GA 30322 USA; 2 Division of Pulmonary, Allergy and Critical Care Medicine, Department of Medicine, Emory University School of Medicine, Atlanta, GA 30322 USA

**Keywords:** Animal disease models, Drosophila

## Abstract

Acetaminophen is the most common cause of acute drug-induced liver injury in the United States. However, research into the mechanisms of acetaminophen toxicity and the development of novel therapeutics is hampered by the lack of robust, reproducible, and cost-effective model systems. Herein, we characterize a novel *Drosophila*-based model of acetaminophen toxicity. We demonstrate that acetaminophen treatment of *Drosophila* results in similar pathophysiologic alterations as those observed in mammalian systems, including a robust production of reactive oxygen species, depletion of glutathione, and dose-dependent mortality. Moreover, these effects are concentrated in the *Drosophila* fat body, an organ analogous to the mammalian liver. Utilizing this system, we interrogated the influence of environmental factors on acetaminophen toxicity which has proven difficult in vertebrate models due to cost and inter-individual variability. We find that both increasing age and microbial depletion sensitize *Drosophila* to acetaminophen toxicity. These environmental influences both alter oxidative stress response pathways in metazoans. Indeed, genetic and pharmacologic manipulations of the antioxidant response modify acetaminophen toxicity in our model. Taken together, these data demonstrate the feasibility of *Drosophila* for the study of acetaminophen toxicity, bringing with it an ease of genetic and microbiome manipulation, high-throughput screening, and availability of transgenic animals.

## Introduction

The FDA estimates that acetaminophen (APAP) is the most widely used drug in the United States, with approximately 28 billion doses purchased per year^1^. As a consequence, APAP overdose is the leading cause of drug-induced liver injury in the United States^2^. Upon ingestion, APAP is absorbed in the intestine and travels to the liver via the portal circulation where it is rapidly glucuronidated or sulfonylated allowing for its harmless excretion^3^. If the liver is exposed to an excess of APAP, however, these detoxification processes are overwhelmed, and APAP is instead metabolized by the cytochrome P450 enzyme CYP2E1 into the highly reactive and toxic metabolite NAPQI^4^. NAPQI rapidly forms adducts with critical cellular proteins, impairs mitochondrial membrane integrity, and results in a profound production of reactive oxygen species (ROS) within hepatocytes^5^. If these ROS overwhelm the cellular antioxidant response, cell death and tissue necrosis occurs, resulting in severe hepatotoxicity that can lead to death^6^.

Despite the clinical importance of APAP overdose, treatment remains limited^7^. Current therapies include limiting absorption of APAP by administering activated charcoal^8–10^, replenishing glutathione stores by administering the glutathione precursor *n*-acetylcysteine (NAC)^11,12^, the JNK/CYP2E1 inhibitor fomepizole (^13^), and supportive care. A complicating factor in clinical trials has been the significant inter-individual variability in susceptibility to APAP toxicity^14^. It has been proposed that this variability is due to genetic differences (i.e. altered expression of Cyp2e1 or conjugation enzymes^15,16^) or environmental exposures (alcohol^17^, drugs^14,18^, or microbiome composition^19^). However, investigation of these factors in preclinical studies is limited due to the time and expense of generating transgenic mice and the variability of the currently available rodent models^20^.

Herein, we present a novel, highly reproducible, *Drosophila*-based system for studying acetaminophen toxicity. Our data demonstrate that acetaminophen accumulates in *Drosophila*, resulting in the generation of ROS in the fat body (an organ analogous to the mammalian liver^21^), a rapid depletion of systemic glutathione, and subsequent mortality. We utilize this system to investigate the effect of the microbiome and aging on APAP toxicity, two variables that have proven difficult to definitively investigate in human or murine systems. Our data demonstrate that the presence of the microbiome is protective in the context of APAP toxicity and that advanced age may play a significant role in susceptibility to drug induced liver injury. Finally, as both increasing age and germ-free conditions have been associated with a decline *Drosophila* antioxidant responses^22,23^, we defined the requirement of the antioxidant response system in our model and find, in agreement with vertebrate studies, that genetic and pharmacologic manipulations of antioxidant response pathways results in altered sensitivity to APAP toxicity^24,25^.

## Results

### Acetaminophen accumulates and results in dose-dependent mortality in WT Drosophila

**Figure 1:**
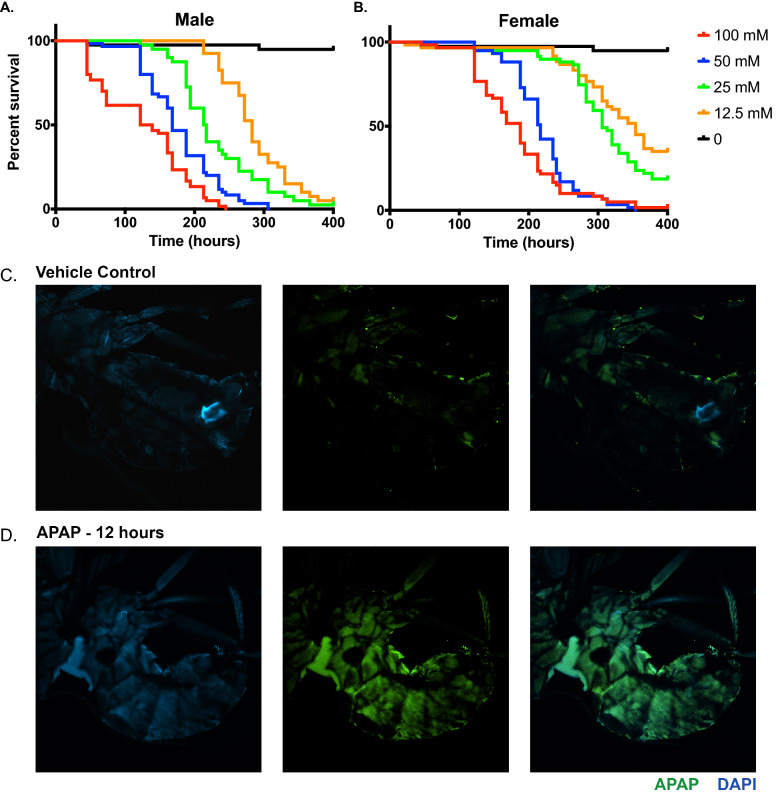
Acetaminophen toxicity and accumulation in adult *Drosophila*. Mortality observed due to APAP exposure in 5-day old w^1118^ (**A**) male and (**B**) female *Drosophila*. Log-Rank *p* = 0.0001, *n* = 60. CLARITY staining for DAPI and anti-APAP antibodies in *Drosophila* treated for 12 h with (**C**) vehicle and (**D**) APAP.

To determine the effect of acetaminophen on adult *Drosophila*, we assessed the viability of 5-day old male (Fig. 1A) and female (Fig. 1B) wild-type flies exposed to APAP at a concentration of 100 mM, 50 mM, 25 mM, 12.5 mM, or 0 mM (vehicle control). APAP exposure resulted in a dose-dependent mortality in both male and female *Drosophila*, with female flies displaying increased resistance to APAP relative to males at all doses. Intriguingly, this effect required continuous exposure to APAP, as animals fed APAP overnight (to simulate a brief/singular dose) showed a mild reduction in survival, but the reduction in survival was not as significant as those who experienced continuous exposure (Supplemental Fig. 1).

To confirm that ingested acetaminophen accumulated in *Drosophila* tissues, we performed immunofluorescence on whole-mounted treated and untreated *Drosophila* adults using an anti-acetaminophen antibody, which recognizes native acetaminophen and acetaminophen-adducts in tissues ^26,27^. Vehicle-fed *Drosophila* show very little background APAP immunoreactivity at 12 h (Fig. 1C), whereas APAP fed adults show increased APAP immunoreactivity, particularly in the abdomen (Fig. 1D).

### APAP administration increases ROS in the drosophila fat body and depletes systemic glutathione

In the mammalian liver, APAP is bio-converted into the toxic metabolite NAPQI by the Cytochrome P450 enzyme CYP2E1^4^. NAPQI is highly reactive and rapidly conjugates with critical cellular enzymes, interfering with normal cellular function and destabilizing the mitochondrial membrane^5^. Due to its impact on mitochondrial function, a hallmark of APAP hepatotoxicity is the robust production of reactive oxygen species (ROS)^28^.

**Figure 2:**
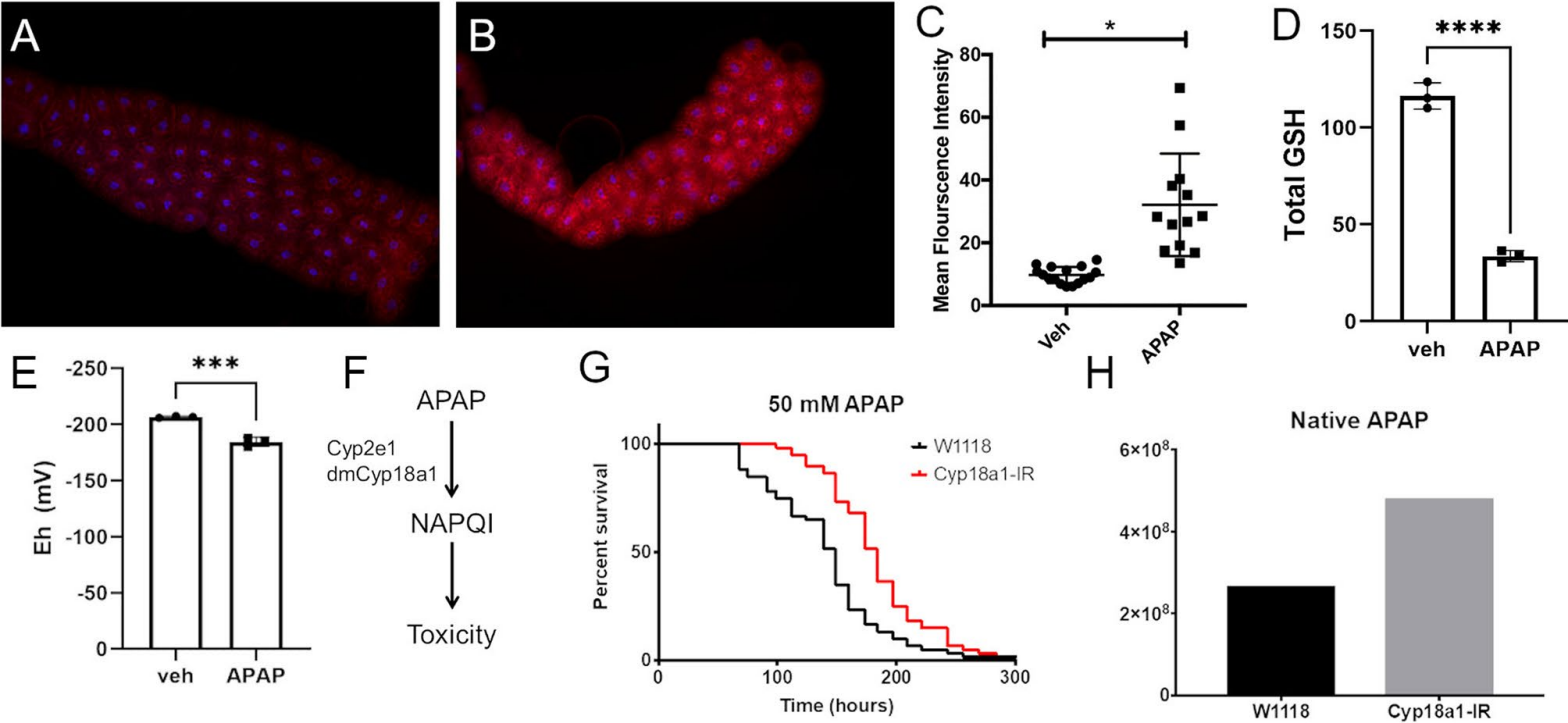
APAP induces ROS, depletes glutathione, and requires Cyp18a1 bioconversion for toxicity in *Drosophila* fat body. Representative fluorescent images of fat body of early third instar *Drosophila* larvae after 4 h of feeding the ROS sensitive dye HydroCy3 and either (**A**) PBS or (**B**) 100 mM APAP in PBS. (**C**) Quantification of fluorescence intensity from imaged fat bodies. *p < 0.05 as determined by t-test. *n* = 13 larvae per condition. (**D**) Total glutathione content of whole *Drosophila* treated with either vehicle control or 100 mM APAP for 3 days. ****p < 0.0001 as determined by t-test; *n* = 3. (**E**) Redox potential of whole *Drosophila* after 3 days of treatment with either vehicle control or 100 mM APAP for 3 days. ***p < 0.001 as determined by t-test; *n* = 3 (**F**) Schematic of APAP metabolism and mechanism of toxicity. (**G**) Effect of fat body-specific depletion of CYP18A1 on survival of *Drosophila* treated with 50 mM APAP. Log-Rank test *p* < 0.0001, *n* = 60. (H) Measurement of native acetaminophen from whole flies using mass spectrometry, *n* = 1.

To determine if APAP ingestion resulted in increased ROS in the *Drosophila* fat body, we stained APAP-treated and control early third-instar larvae with HydroCy3, a ROS sensitive dye that fluoresces in the presence of superoxide radicals^29^. Control larvae showed minimal ROS production in the fat body (Fig. 2A). In contrast, APAP feeding significantly increased ROS in the fat body (Fig. 2B,C).

NAPQI is rapidly conjugated to glutathione, limiting its toxicity while depleting cellular glutathione stores^14,30^. Thus, we assessed the effect of APAP feeding on systemic glutathione levels and redox potential (Fig. 2D,E). APAP feeding resulted in a significant decrease in total glutathione levels relative to vehicle control, with greater than a 70% reduction in total GSH levels after 3 days of exposure (116.42 ± 5.55 uM GSH for vehicle control vs 33.67 ± 2.35 for APAP treated). Similarly, redox potential was significantly reduced in APAP-treated animals (-206.77 ± 0.65 mV for vehicle control vs -184.52 ± 3.34 mv for APAP treated) consistent with increased oxidant exposure.

### Fat body-specific depletion of CYP18A1 attenuates APAP toxicity.

In the mammalian system, APAP requires bioconversion by CYP2E1 for toxicity^31^. Using the DRSC Integrative Ortholog Prediction Tool^32^, we identified the *Drosophila* gene *cyp18a1* as a putative orthologue of CYP2E1 (Fig. 2F). To test the requirement of CYP18A1 in our system, we depleted CYP18A1 using the GAL4/UAS system and assessed the response to APAP treatment. Depletion of CYP18A1 in the fat body rendered these *Drosophila* resistant to APAP-induced mortality (Fig. 2G), in agreement with genetic studies in murine models ^31^. Consistent with a role in APAP bioconversion, metabolomic analysis of CYP18A1-depleted larvae revealed an increase in native acetaminophen relative to wild-type larvae (Fig. 2H). Taken together, these data demonstrate that CYP-driven bioconversion of APAP occurs in our model and augments mortality, thus recapitulating similar findings described in vertebrate models^31^.

### APAP administration promotes apoptotic-response and regenerative in the adult fat body

**Figure 3:**
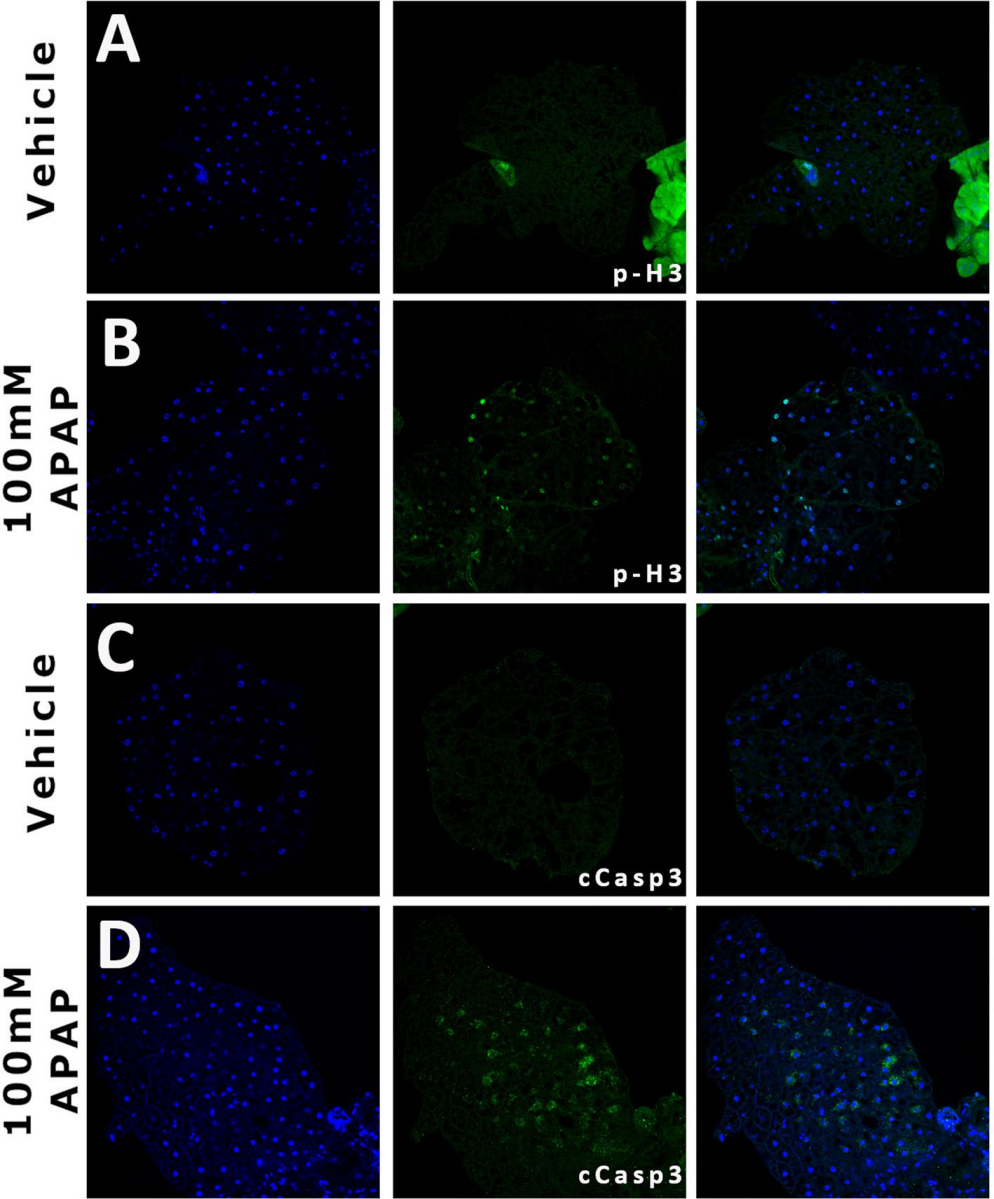
APAP exposure induces regenerative and apoptotic responses in the adult fat body. Representative images of adult *Drosophila* fat bodies from vehicle-treated (**A** and **C**) and 100 mM-treated (**B** and **D**) animals stained for p-H3 (**A** and **B**) and cCasp3 (**C** and **D**). APAP and/or vehicle exposure was 4 days. p-H3 = phosophorylated Histone 3; cCasp3 = cleaved caspase 3.

In vertebrate systems, APAP administration is associated with massive necrosis, possible apoptosis, and a prompt regenerative response ^33,34^. We examined the adult fat bodies in treated and untreated animals to see whether similar responses are present in our models. In untreated animals, cleaved caspase-3 (cCasp-3) expression (a marker of cell death induction) is not observed; however, on day 4 animals treated with 100 mM APAP there is patchy cCasp-3 expression (Fig. 3C,D). Similarly, analysis of phosphorylated Histone H3 (a marker of cellular division) shows increased expression in 4-day treated animals that is not observed in control animals (Fig. 3A,B). These animals suggest that APAP-mediated injury in the Drosophila fat body is sufficient to induce cell death and a regenerative response.

### Effects of environmental factors on APAP-induced mortality

**Figure 4:**
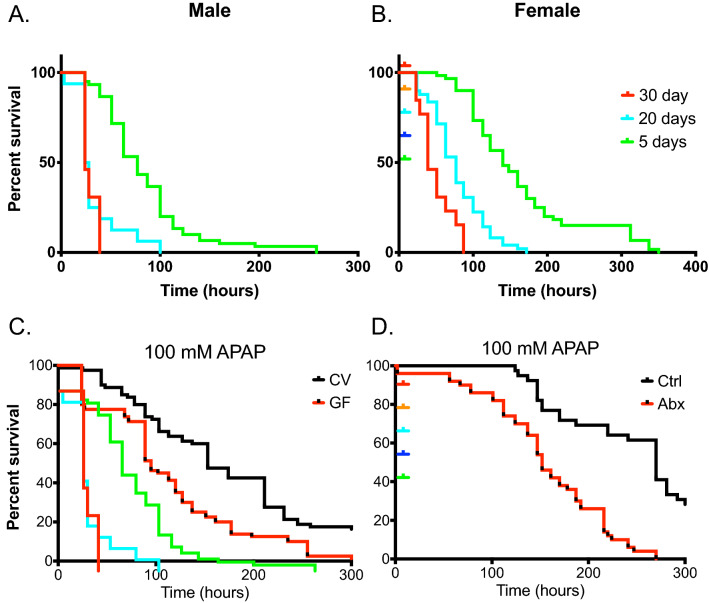
Effects of age and the microbiome on APAP-induced mortality. Susceptibility of 5, 20, and 30-day old (**A**) male and (**B**) female flies to APAP induced mortality. Log-Rank test *p* < 0.0001, *n* = 60. (**C**) Survival of germ-free (GF) and conventional (CV) *Drosophila* exposed to 100 mM APAP. (**D**) Effect of antibiotic-mediated depletion of the microbiome on susceptibility to APAP toxicity. Log-Rank test *p* < 0.0001, *n* = 60.

The above data demonstrate that our *Drosophila*-based system recapitulates studies performed in mice and demonstrates a similar pathologic mechanism for acetaminophen toxicity in *Drosophila*. To extend these findings, we next utilized our model to pilot studies that are more difficult, time-consuming, and expensive in vertebrate models, including examining the effects of aging and microbiome. Few human or murine studies exist regarding the influence of aging on susceptibility to drug induced liver injury^35^, which is often complicated by confounding factors including polypharmacy and genetics, among others. To determine if aging altered susceptibility to APAP, we characterized the mortality in 5, 10, and 30-day old flies with and without APAP treatment. We find that wild-type *Drosophila* show a striking age-dependent increase in APAP-induced mortality (Fig. 4A,B). Moreover, this effect is most pronounced in the oldest flies (30 days old at time of APAP administration) and is observed in both males and females (Fig. 4A,B).

It has been proposed that the microbiome may modify susceptibility to APAP toxicity in humans and thereby explain interindividual susceptibility to drug-induced liver injury^19^. Studies investigating the severity of APAP hepatotoxicity in GF vs conventional mice have been inconclusive^36^. To test the role of the microbiome in our system, we treated 5-day old germ-free or conventional *Drosophila* with 100 mM APAP. Conventionalized animals show significant protection to APAP induced toxicity compared to their germ-free counterparts (Fig. 4C). Moreover, antibiotic-treated conventionalized *Drosophila* adults (microbiome-depleted) have significantly reduced protection to APAP-induced mortality (Fig. 4D).

### Genetic and pharmacologic modification of antioxidant response pathways alter APAP toxicity in Drosophila

**Figure 5:**
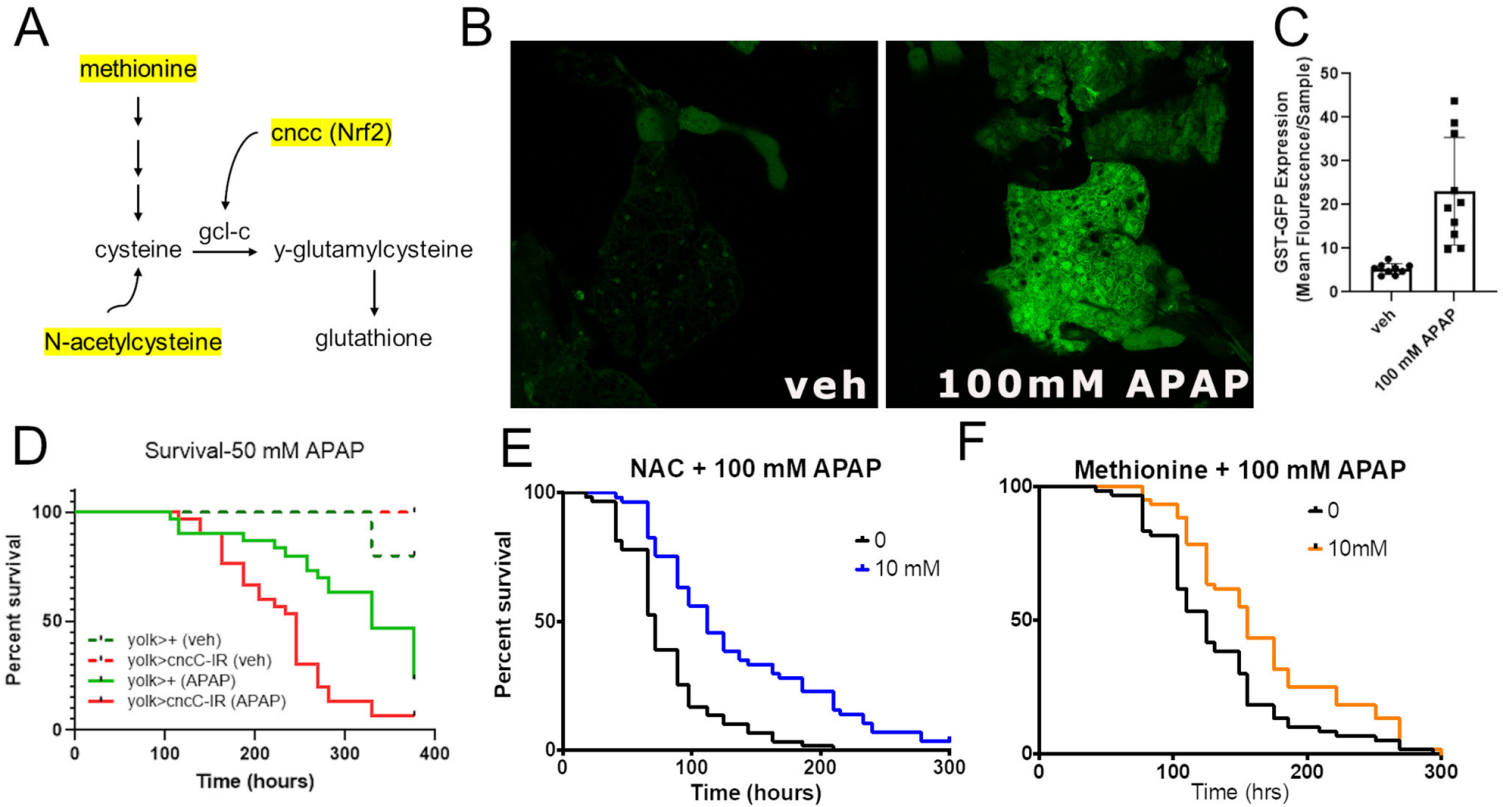
Genetic and pharmacologic modification of antioxidant response pathways alter APAP toxicity in *Drosophila*. (**A**) Schematic representation of glutathione generation in metazoans, highlighting the role of Nrf2/CncC, N-acetylcystein, and methione (in yellow). (**B**) Representative images and (**C**) quantification of mean GFP fluorescence in control and 100 mM APAP treated ARE-GST-GFP animals. (**D**) Survival of vehicle treated and 50 mM APAP-treated control and experimental animals, in which dNrf2 knockout (cnccIR) is depleted in the *Drosophila* fat body using the fat body driver Yolk-Gal4. Log-Rank test *p* < 0.0001, *n* = 60. (**E**) Effect of 100 mM N-acetylcysteine supplementation on APAP toxicity. (**F**) Effect of 10 mM Methionine supplementation of APAP toxicity. Log-Rank test *p* < 0.0001, *n* = 60.

Several signaling pathways are linked to aging and the microbiome, including altered JNK-signaling and anti-oxidant response pathways. We first tested the requirement of JNK-signaling in our system. Expression of a dominant-negative form of the terminal Jun Kinase (termed *basket*), which blocks JNK-signaling in tissues^37^, produced only mild effects on APAP-induced mortality (Supplemental Fig. 2). To test the role of antioxidant signaling, we examined the activity and requirement of components of the anti-oxidant response pathway (Fig. 5A), including the Nrf2 ortholog Cap n’ collar (CncC)^22^. Examination of the GST-GFP reporter, which contains multiple anti-oxidant response elements (ARE) and is activated in oxidized tissue^38^, shows robust activations in APAP-exposed animals (Fig. 5B,C); importantly, this reporter is not activated when the antioxidant response element is removed (Supplemental Fig. 3). Next, we depleted CncC in the *Drosophila* fat body and assessed their susceptibility to APAP-induced mortality. In agreement with studies conducted in mice, CncC knockdown augmented APAP toxicity and increased mortality (Fig. 5D).

N-acetyl cysteine (NAC) is the current standard of care for APAP overdose^12^, which is It is believed to have benefit in APAP hepatotoxicity by increasing the availability of the glutathione precursor cysteine, thereby driving glutathione synthesis. To test its efficacy in the *Drosophila* model, we treated *Drosophila* concurrently with both NAC and APAP. At 10 mM, NAC feeding significantly protected against APAP induced mortality relative to vehicle control (Fig. 5E). Another small molecule that works to increase glutathione and has shown efficacy in human disease is the amino acid methionine^39^. In agreement with these studies, 10 mM methionine treatment attenuated APAP toxicity in *Drosophila* (Fig. 5F).

## Discussion

Acetaminophen overdose is the most common cause of drug-induced liver injury in the United States^2^. While some overdoses are intentional, many result from the inadvertent combination of one of innumerable acetaminophen containing drugs. Despite its ubiquity, acetaminophen can cause significant hepatic injury at relatively low doses, and current therapy is limited and primarily supportive^12^.

Herein, we describe the use of *Drosophila* melanogaster as a model for APAP-induced oxidative liver injury. Our data demonstrate that APAP accumulates within flies, results in reactive oxygen species generation within the fat body, glutathione depletion, cell death, and mortality in a dose-dependent manner. Furthermore, APAP toxicity in *Drosophila* is modified by genetic and pharmacologic manipulations in anti-oxidant pathways that have been validated in both mice and humans. Finally, we utilized our model to determine the impact of antibiotic use and age on APAP toxicity, which to our knowledge have not been previously described.

The use of *Drosophila* bring to the study of oxidative liver injury many advantages, including ease of genetic manipulation, availability of genetic mutants and constructs, ease of germ-free generation, relatively short lifespan, amenability to high-throughput screening, and overall cost relative to murine models^40^. However, some limitations should be noted. First, whereas the human and murine pathology emanates from a single supra-therapeutic dose of APAP, we only observed significant mortality after continuous ad-lib exposure. This is likely secondary to the well-documented pharmacokinetic properties of *Drosophila* (and other invertebrate model system) which are distinct from vertebrate counterparts, limiting the ability to dosing studies, etc.^41^. The fat body, while analogous, lacks the structural organization and cellular make-up of the mammalian liver. Moreover, *Drosophila* lack a portal circulation that moves absorbed intestinal metabolites directly to the fat body. Instead, the fat body and other systemic organs are bathed in “hemolymph.” Therefore, it is possible that absorbed acetaminophen may impact other systemic organs as well as the fat body, whereas in mammals APAP toxicity is fairly specific to the liver. Finally, *Drosophila* do not have an adaptive immune system, which complicates both the study of tissue restitution after injury and the underlying crosstalk with intestinal microbes.

Despite these limitations, the use of *Drosophila* holds great potential for the study of APAP overdose and potentially other forms of drug-induced liver injury. Genetic screens may help identify previously uncharacterized genetic modifiers of susceptibility. High-throughput compound screens may yield new therapeutics. Moreover, as to our knowledge these data are the first to show a proliferative response in the *Drosophila* adult fat body in response to injury, these data provide evidence for a new modality to study liver and tissue regeneration in *Drosophila*. Germ-free and monocolonization studies may provide some insight into the opaque and complex bidirectional signaling between the microbiome and systemic organs. The model presented here, therefore, shows great promise for the advancement of the study of drug-induced liver injury and the study of tissue regeneration in metazoans.

## Methods

### Drosophila

*Drosophila* were maintained on standard diets at 25 °C with 12-h light/dark cycles (7 am–7 pm). Unless otherwise stated, WT flies are of the w^1118^ background. Tissue specific genetic manipulations were performed using the Gal4-UAS system described previously^42^. Briefly, virgin Yolk-Gal4 or R4-Gal4 *Drosophila* were crossed with male w^1118^ (control), UAS-CNCC-IR (dNrf2 knockdown), UAS-CNCC (dNrf2 overexpression), or UAS-Cyp18a1-IR (Cyp18a1 knockdown). Offspring were collected 5 days post-eclosure and used for experimentation.

### Acetaminophen Treatment

Acetaminophen was dissolved in 5% sucrose and 1% agar and dispensed in into empty vials. 20 flies were transferred to each vial and mortality recorded approximately every 12 h. Vehicle control vials contained only 5% sucrose and 1% agar. N-acetylcysteine and methionine (Sigma-Aldrich) were dissolved in the APAP media at the indicated concentrations.

### Immunofluorescence

Immunostaining and confocal microscopy performed using standard procedures. Briefly, adult fat bodies were dissected in 1× phosphate buffered saline (PBS), fixed 20 min in 4% paraformaldehyde at room temperature (RT), rinsed 5× in 1× PBS, then permeabilized for 30 min at RT in 1xPBS + 0.3% Triton X-100. Samples were then rinsed 2× in 1 × PBS, resuspended in primary antibody diluted (according to manufacturer instructions) in 1 × PBS + 0.1% Triton X-100 plus in 10% normal goat serum (NGS; Jackson Laboratories). Samples were incubated overnight at 4C, rinsed 5× in 1xPBS + 0.1% Triton X-100, then incubated overnight at 4C in secondary antibody + 10% NGS in 1xPBS + 0.1% Triton X-100. Following 3× washes in 1xPBS + 0.1% Triton X-100, discs were mounted in Vectashield containing DAPI (Vector Labs, H-2000). Images were obtained on an Olympus FV1000 confocal microscope and processed with FIJI and GIMP software. Primary antibodies includes rabbit anti-phospho-H3 (1:100, Cell signaling, cat#9701), rabbit anti-cleaved caspase 3 (1:100, Cell signaling, cat#9661), sheep anti-acetaminophen primary antibody (1:250, Bio-Rad, cat#0016-0104). Secondary antibodies include goat anti rabbit IgG Alexa Fluor 488 (1:100, Jackson, ab2338046) and donkey anti-sheep IgG Alexa Fluor 488 (1:500, Abcam, ab150177).

The tissue clearing CLARITY protocol was used to visualize epitope distribution in whole animals for acetaminophen immunofluorescence. Briefly, whole *Drosophila* were fixed in 4% PFA for 12 h at 4 °C, then rinsed 3× PBS for 3 h at RT with gentle shaking, and then immersed in FlyClear Solution-1 (8% THEED (2,2′,2″,2″′- (Ethylenedinitrilo)-tetraethanol) (Sigma-Aldrich), 5% Triton X-100 (Sigma-Aldrich) and 25% Urea (Fisher Scientific, in PBS) for 14 days at 37 °C, as described previously ^26^. Cleared *Drosophila* tissues were rinsed 3 × in PBS with gentle shaking for 12 h at RT, then immersed in blocking solution and processed as outlined above.

### HydroCy3 Analysis

Early third-instar *Drosophila* larvae were fed for 4 h on either PBS (vehicle control) or 100 mM APAP along with HydroCy3 (ROSstar 550, Li-Cor). Fat bodies were dissected, whole-mounted on glass microscope slides and imaged using Nikon eclipse 80i microscope fitted with a R1 Retiga Q Imaging camera. Quantification of fluorescence intensity performed using FIJI software (NIH).

### Glutathione measurements

Total glutathione levels and redox potential were determined using HPLC to determine glutathione metabolites following derivatization with dansyl chloride. Briefly, cohorts of 30–50 adult flies (approximately 50 mg of fresh tissue) were collected in Eppendorf tubes containing and transferred to 500 ul ice-cold 50 g/l perchloric acid solution containing 0.2 M boric acid and 10 uM gamma-Glu-Glu on ice. Flies were homogenized for 15 s using a Teflon micropestle and the homogenate centrifuged at 14,000 g for 2 min. Aliquots of 300 ul of the supernatant were transferred to fresh tubes for further analysis, while the remaining supernatant fluid was discarded and the protein pellet resuspended in 200 ul of 1 N NaOH and analyzed for protein quantification using the BioRad DC Assay with BSA as a standard. Samples were stored at − 80 °C until they were derivatized with 60 ul of 7.4 mg/ml sodium iodoacetic acid. The pH was adjusted to 8.8–9.2 with 1 M KOH saturated K3B4O7 and 300 ul of 20 mg/ml dansyl chloride, followed by incubation in the dark at room temperature 24 h. 500 ul of chloroform is then added to each of the samples to extract acetone and “free” dansyl chloride. Analysis by HPLC with fluorescence detection was performed as previously described^43,44^. Concentrations of thiols and disulfides were determined by integration relative to an internal standard^45^. Redox potential (Eh) was calculated from the cellular GSH and GSSG concentrations using the Nernst equation as described^46^. A less negative value indicates a more oxidized redox state.

### Generation of germ-free flies

Adult flies were placed in vials with fresh food and left overnight (8–14 h). The vials were emptied and ~ 5 mL dH2O was added to each vial. A paintbrush was used to suspend the remaining eggs in the dH2O, then the dH2O and eggs were poured into 90um cell strainers. In an aseptic environment, the cell strainers were placed in 50% bleach for 10 min, then transferred into sterile dH2O for 1 min three times consecutively. Using a sterile scalpel and forceps, the bottoms of the cell strainers were removed and placed in new, autoclaved vials with germ-free fly food. To verify the absence of bacteria, flies from two different germ-free vials were crushed in ~ 200uL of sterile dH2O in an aseptic environment, then plated on blood agar. The blood agar plate was left overnight at 37 degrees Celsius. No bacteria were observed growing on the plate.

### Antibiotic treatment

5-day old *Drosophila* were transferred to media containing 5% sucrose, 1% agar, 100 ug/mL ampicillin, and 50 ug/mL streptomycin (Sigma-Aldrich). 3 days later, *Drosophila* were transferred to fresh vials containing acetaminophen without antibiotics and mortality assessed.

## Supplementary Information

Supplementary Information.

## Data Availability

The datasets used and/or analysed during the current study available from the corresponding author on reasonable request.

## References

[1] Bunchorntavakul C, Reddy KR (2018). Acetaminophen (APAP or N-Acetyl-p-Aminophenol) and acute liver failure. Clin. Liver Dis..

[2] Yoon E (2016). Acetaminophen-Induced Hepatotoxicity: a Comprehensive Update. J. Clin. Transl. Hepatol..

[3] Forrest JA, Clements JA, Prescott LF (1982). Clinical pharmacokinetics of paracetamol. Clin. Pharmacokinet.

[4] Manyike PT (2000). Contribution of CYP2E1 and CYP3A to acetaminophen reactive metabolite formation. Clin. Pharmacol. Ther..

[5] Gemborys MW, Mudge GH, Gribble GW (1980). Mechanism of decomposition of N-hydroxyacetaminophen, a postulated toxic metabolite of acetaminophen. J. Med. Chem..

[6] Nelson SD (1990). Molecular mechanisms of the hepatotoxicity caused by acetaminophen. Semin Liver Dis.

[7] Stine JG, Lewis JH (2016). Current and future directions in the treatment and prevention of drug-induced liver injury: a systematic review. Expert Rev. Gastroenterol. Hepatol..

[8] Gazzard BG (1974). Charcoal haemoperfusion for paracetamol overdose. Br. J. Clin. Pharmacol..

[9] Underhill TJ, Greene MK, Dove AF (1990). A comparison of the efficacy of gastric lavage, ipecacuanha and activated charcoal in the emergency management of paracetamol overdose. Arch. Emerg. Med..

[10] Chiew AL (2017). Massive paracetamol overdose: an observational study of the effect of activated charcoal and increased acetylcysteine dose (ATOM-2). Clin. Toxicol. (Phila.).

[11] Rumack BH (1981). Acetaminophen overdose. 662 cases with evaluation of oral acetylcysteine treatment. Arch. Intern. Med..

[12] Chiew AL (2018). Interventions for paracetamol (acetaminophen) overdose. Cochrane Database Syst. Rev..

[13] Link SL (2022). Fomepizole as an adjunct in acetylcysteine treated acetaminophen overdose patients: a case series. Clin. Toxicol. (Phila.).

[14] McGill MR, Jaeschke H (2013). Metabolism and disposition of acetaminophen: Recent advances in relation to hepatotoxicity and diagnosis. Pharm. Res..

[15] Critchley JA (1986). Inter-subject and ethnic differences in paracetamol metabolism. Br. J. Clin. Pharmacol..

[16] Zhao L, Pickering G (2011). Paracetamol metabolism and related genetic differences. Drug Metab. Rev..

[17] Schmidt LE, Dalhoff K, Poulsen HE (2002). Acute versus chronic alcohol consumption in acetaminophen-induced hepatotoxicity. Hepatology.

[18] Abebe W (2002). Herbal medication: potential for adverse interactions with analgesic drugs. J. Clin. Pharm. Ther..

[19] Klaassen CD, Cui JY (2015). Review: Mechanisms of how the intestinal microbiota alters the effects of drugs and bile acids. Drug Metab. Dispos..

[20] Mossanen JC, Tacke F (2015). Acetaminophen-induced acute liver injury in mice. Lab Anim.

[21] Baker KD, Thummel CS (2007). Diabetic larvae and obese flies-emerging studies of metabolism in Drosophila. Cell Metab..

[22] Sykiotis GP, Bohmann D (2010). Stress-activated cap'n'collar transcription factors in aging and human disease. Sci. Signal.

[23] Jones RM (2015). Lactobacilli Modulate Epithelial Cytoprotection through the Nrf2 Pathway. Cell Rep..

[24] Chan K, Han XD, Kan YW (2001). An important function of Nrf2 in combating oxidative stress: detoxification of acetaminophen. Proc. Natl. Acad. Sci. USA.

[25] Saeedi BJ (2020). Gut-resident lactobacilli activate hepatic nrf2 and protect against oxidative liver injury. Cell Metab..

[26] Pende M (2018). High-resolution ultramicroscopy of the developing and adult nervous system in optically cleared Drosophila melanogaster. Nat. Commun..

[27] Scheiermann P (2013). Application of interleukin-22 mediates protection in experimental acetaminophen-induced acute liver injury. Am. J. Pathol..

[28] Jaeschke H, Xie Y, McGill MR (2014). Acetaminophen-induced liver injury: from animal models to humans. J. Clin. Transl. Hepatol..

[29] Saeedi BJ, Chandrasekharan B, Neish AS (2019). Hydro-Cy3-mediated detection of reactive oxygen species in vitro and in vivo. Methods Mol. Biol..

[30] Mitchell JR (1973). Acetaminophen-induced hepatic necrosis. IV. Protective role of glutathione. J. Pharmacol. Exp. Ther..

[31] Lee SS (1996). Role of CYP2E1 in the hepatotoxicity of acetaminophen. J. Biol. Chem..

[32] Hu Y (2011). An integrative approach to ortholog prediction for disease-focused and other functional studies. BMC Bioinf..

[33] Akakpo JY, Ramachandran A, Jaeschke H (2020). Novel strategies for the treatment of acetaminophen hepatotoxicity. Expert Opin. Drug Metab. Toxicol..

[34] Ge Z (2019). Tempol protects against acetaminophen induced acute hepatotoxicity by inhibiting oxidative stress and apoptosis. Front. Physiol..

[35] Mian P (2018). Paracetamol in older people: Towards evidence-based dosing?. Drugs Aging.

[36] Possamai LA (2015). The role of intestinal microbiota in murine models of acetaminophen-induced hepatotoxicity. Liver Int..

[37] Igaki T (2002). Eiger, a TNF superfamily ligand that triggers the Drosophila JNK pathway. EMBO J..

[38] Sykiotis GP, Bohmann D (2008). Keap1/Nrf2 signaling regulates oxidative stress tolerance and lifespan in Drosophila. Dev. Cell.

[39] Prescott LF (1976). Cysteamine, methionine, and penicillamine in the treatment of paracetamol poisoning. Lancet.

[40] Ugur B, Chen K, Bellen HJ (2016). Drosophila tools and assays for the study of human diseases. Dis. Model Mech..

[41] Pandey UB, Nichols CD (2011). Human disease models in Drosophila melanogaster and the role of the fly in therapeutic drug discovery. Pharmacol. Rev..

[42] Duffy JB (2002). GAL4 system in Drosophila: A fly geneticist's Swiss army knife. Genesis.

[43] Jones DP (1998). Glutathione measurement in human plasma. Evaluation of sample collection, storage and derivatization conditions for analysis of dansyl derivatives by HPLC. Clin. Chim. Acta.

[44] Miller LT (2002). Oxidation of the glutathione/glutathione disulfide redox state is induced by cysteine deficiency in human colon carcinoma HT29 cells. J. Nutr..

[45] Jones DP (2000). Redox state of glutathione in human plasma. Free Radic. Biol. Med..

[46] Kirlin WG (1999). Glutathione redox potential in response to differentiation and enzyme inducers. Free Radic. Biol. Med..

