# Supplementary material for: Oxidative stress mediates end-organ damage in a novel model of acetaminophen-toxicity in Drosophila
Source: Sci Rep. 2022 Nov 11;12:19309. doi: 10.1038/s41598-022-21156-w (PMC9652370; doi:10.1038/s41598-022-21156-w)
Supplement: Supplementary file 1 — Supplementary Information. [file 41598_2022_21156_MOESM1_ESM.docx]

| Description | Title | Page |
| --- | --- | --- |
| Supplemental Figure 1 | Continuous exposure to APAP shows more dramatic effects on survival than overnight exposure | 2 |
| Supplemental Figure 2 | Genetic modification of JNK-signaling does not significantly alter APAP toxicity in *Drosophila* | 3 |
| Supplemental Figure 3 | Genetic modification of JNK-signaling does not significantly alter APAP toxicity in *Drosophila* | 4 |

**Supplemental Figure 1: Continuous exposure to APAP shows more dramatic effects on survival than overnight exposure.**

Survival of wild-type *Drosophila* adults (w^1118^) who were either vehicle-treated (green), treated with 100 mM APAP for their entire lives (red), or were briefly exposed to 100 mM APAP overnight (orange).


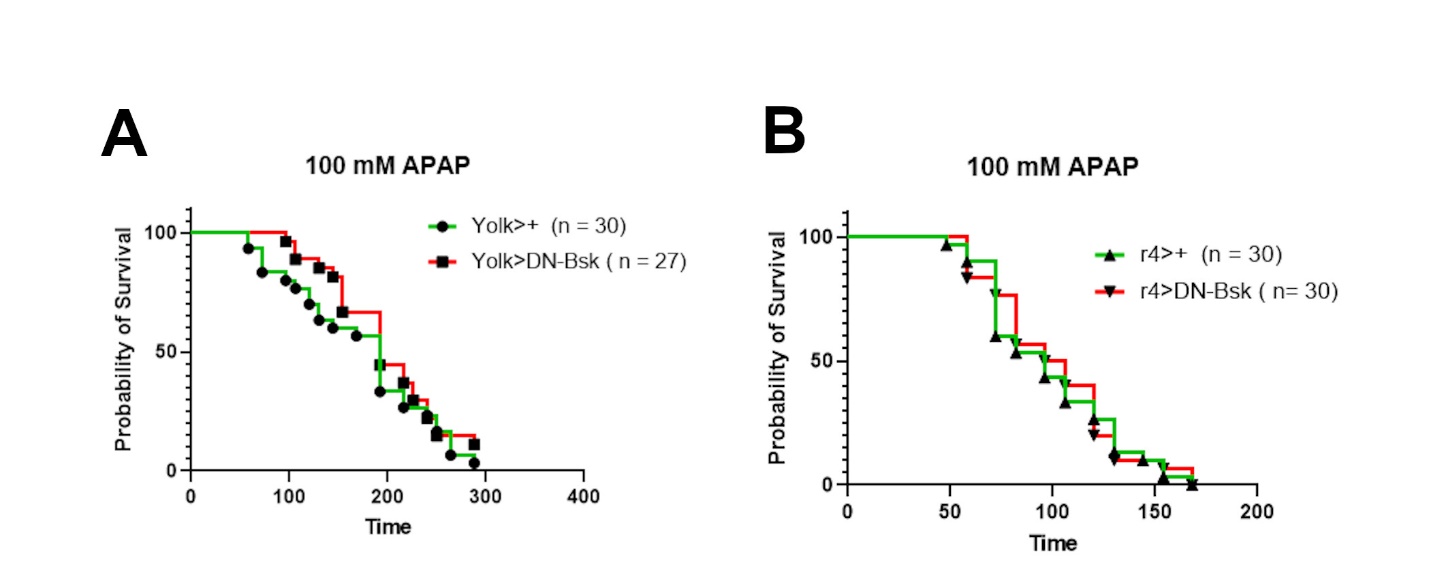


Supplemental Figure 2: Genetic modification of JNK-signaling does not significantly alter APAP toxicity in *Drosophila*.

Survival of 100 mM APAP treated animals in control (green) *Drosophila* adults and organisms in which that transgene DN-Bsk was expressed in the fat body (red lines) using the tissue specific drivers (A) Yolk-Gal4 and (B) r4-Gal4. Log Rank test did not show significant differences between curves.


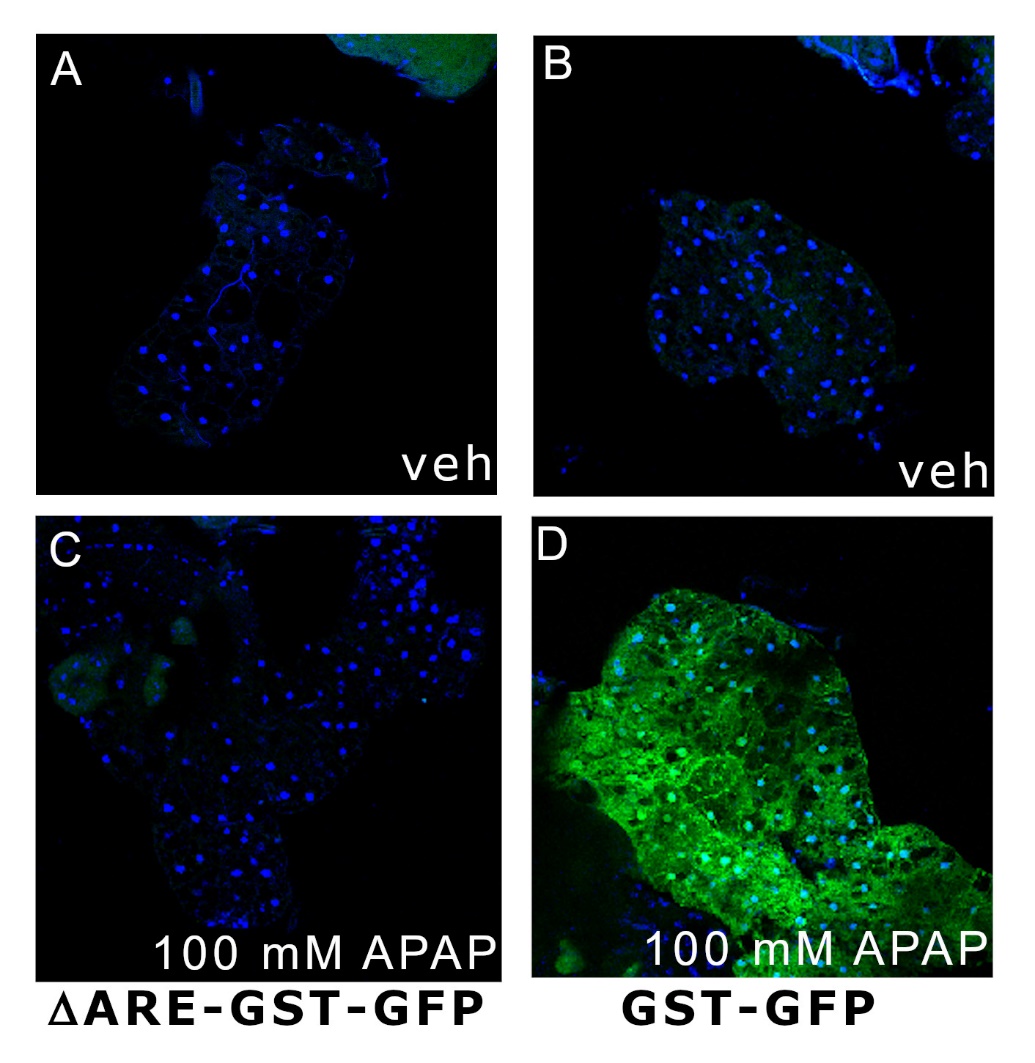


Supplemental Figure 3: Activation of GST-GFP by APAP requires the antioxidant response element.

Representative images of vehicle (A and B) and 100 mM APAP (C and D) treated animals in the background of the GST-GFP reporter (B and D) and one in which the antioxidant response element has been removed (A and C).
